# Notification in Tuberculosis

**Published:** 1903-10-31

**Authors:** 


### The Hospital.

A JOURNAL OF

The Medical Sciences and Hospital Administration.

VOL. XXXV.—NO. 892. OCTOBER 31, 1903.

### NOTIFICATION IN TUBERCULOSIS.

THE *Journal* of the Sanitary Institute for October, just published, contains a highly instructive series of papers, read at the Bradford Congress, on the Notification of Pulmonary Tuberculosis, and a report of an equally instructive discussion which was founded upon them. The papers were by Dr. Newsholme, dealing with the experience of Brighton; by Drs. Kenwood and Gerard C. Taylor, dealing with that of the metropolis, and by Dr. Niven, dealing with that of Manchester; while the discussion served to throw light upon the special requirements and conditions of rural districts. There were marked differences of opinion on the question whether "voluntary" notification would, generally speaking, be sufficient for the purposes which it was intended to fulfil, or whether it should be rendered "compulsory," and, in this regard, the President of the Congress, Professor Clifford Allbutt, rendered good service to the cause of effective sanitation by suggesting that it would be wise to substitute the word "systematic" for "compulsory." With the wisdom born of long experience he recognised that what a thing is called may sometimes be of greater importance than what it is; and the general course of the debate was such as clearly to establish that notification, the more extended and the more complete the better, might be so conducted as to render important service in the control of the disease, and, at the same time, to be free from the principal objections which were raised against it at the time when it was first proposed.

These objections were, it will be remembered, very lucidly set forth by the late Sir Richard Thorne Thorne in his Harben lectures on tuberculosis, and they chiefly depended upon the complete inapplicability to a chronic malady of the measures which, as consequences of notification, are applied to cases of acute zymotic disease. The sufferer from pulmonary tuberculosis, it was said, could not be subjected to the restraints which were applied to the sufferer from scarlet fever; and the only effect of notification would be to place hindrances in the way of his obtaining such work as he might be able to perform, and to render him liable to be shunned throughout the whole progress of a tedious illness alike by his own family and by strangers. As carried into effect at Brighton, however, none of these evil consequences appear to have been produced; and it is manifest that a large amount of indirect protection has been afforded to the community. As soon as possible after notification, the patient is afforded an opportunity of going for a month to a sanatorium, where his stay is not so much intended to be curative as educational. He is made to realise the improvement in his own condition which is brought about by breathing fresh, as opposed to vitiated, air. He is instructed as to the diet best adapted for his requirements. He is taught to be careful about his sputa, and in this respect precept is enforced by the example of those around him, and, if necessary, by the discipline of the establishment. During his absence his home is thoroughly cleansed and disinfected. He returns to it not only in better health, but perfectly conscious of the means by which the improvement has been brought about, and anxious, in the great majority of cases, to continue in the exercise of precautions the efficacy of which he has come to appreciate and to understand. He is provided with a spitting cup and with a supply of Japanese paper handkerchiefs, and, if he be able to resume his employment, it is usually found that he will be careful not to expose others to any avoidable risk of infection. His own disease will probably run its accustomed course, but its prejudicial influence upon the community is likely to be materially diminished.

It is manifest that the educational treatment of the consumptive patient, as thus indicated, may be effectively carried out amid very simple surroundings, and without the aid either of costly buildings or of a numerous staff. Dr. Newsholme obtained the consent of the Brighton Town Council to the admission of four consumptive patients into one of the isolation pavilions of the Borough Fever Hospital, and the experiment was so successful that the number of beds utilised for the purpose was soon increased to 10. Through these it has been found possible to pass from 100 to 120 consumptive patients in the course of the year; and, as the total deaths from the disease in Brighton in 1902 were only 174, as each consumptive patient lives several years, and as those of higher social status are not so likely to be sources of danger to others, it can be confidently hoped that in a few years every consumptive patient in the town will have had a month's practical training in the simple precautions required to prevent him from being a source of danger to others. Dr. Newsholme's experience of the effects has, so far, been highly encouraging. He says that when the patients leave the sanatorium at the end of the month, which is the usual limit of time, they are without exception ardent advocates of the fresh-air *regime*, and that he has not met with one out of the 71 treated up to June last, who has again become careless as to coughing and expectoration. Each patient is informed, on his admission, that a cure is impossible in the time during which he will be treated, but that he will have a thorough holiday and rest under excellent conditions, and will be taught so to manage himself that when he leaves he can, as far as his means permit, continue the treatment, and pursue his daily life without risk to his relatives or his fellow workmen. It is probable that "notification," to be followed by such treatment as this, would be acceptable rather than not to the classes chiefly concerned; and that, by the adoption of a similar plan in other and more populous places, it might be rendered "systematic" even although it were not "compulsory."

